# Role of Laparoscopy in the Management of Acute Surgical Abdomen Secondary to Phytobezoars

**DOI:** 10.7759/cureus.1363

**Published:** 2017-06-17

**Authors:** Abu Baker Sheikh, Aisha Akhtar, Adeel Nasrullah, Shujaul Haq, Haider Ghazanfar

**Affiliations:** 1 Department of Internal Medicine, Shifa International Hospital; 2 Surgery, Texas Tech Health Sciences Center Lubbock

**Keywords:** intestinal obstruction, phytobezoar, laparotomy, laparoscopy, computer tomography, abdominal surgery, acute abdomen

## Abstract

A bezoar is a collection of indigestible material found in the alimentary canal, which can cause mechanical obstruction of the gastrointestinal tract. Phytobezoar is a variant composed of mostly plant material and indigestible fiber. Phytobezoar is a rare cause of small bowel obstruction (SBO) and happens more commonly in patients with risk factors predisposing to impaired gastrointestinal motility. We present a rare case of SBO secondary to phytobezoar in a 60-year-old female patient with type 2 diabetes. There was no prior history of any abdominal surgery. The abdominal computed tomography (CT) scan was inconclusive. Laparoscopy was found to be an effective diagnostic and therapeutic procedure in this patient.

## Introduction

Small bowel obstruction (SBO) is routinely seen in surgical clinics and emergency departments. SBO is a common disease and has a few common etiologies such as adhesions (73.8%) and hernia (18.5%) [1]. Of the rarer causes, one is bezoars. Bezoars account for approximately 2-4% cases of small bowel obstruction and can occur in all age groups [1]. A “bezoar” is a collection of partially digested or undigested contents found in the alimentary canal [2]. It usually forms in the stomach and can cause SBO as it passes through the gastrointestinal (GI) tract. Bezoars can present with a variety of symptoms. Some of these include pain, blood in stool, abdominal discomfort and fullness, anemia, hematemesis, nausea, and vomiting.

Bezoars are classified according to the composition. The four types of bezoars are phytobezoar, composed of vegetable fibers and undigested fruit fibers; trichobezoar, composed of hairs; lactobezoar, made up of milk curd; and pharmacobezoar, composed of different kind of drugs [3]. Phytobezoar is the most common type of bezoar. Peptic ulcer, intake of excessive amounts of indigestible vegetables, impaired gastric motility, history of gastric surgery and medical conditions such as diabetes mellitus and hypothyroidism are the common risk factors for the development of phytobezoars.

Diagnostic laparoscopy has revolutionized the modern surgical practices and has a valid role in diagnosing the pathology in obstructed bowel without increasing morbidity, and it can be used not only as a diagnostic tool but can be used therapeutically as well. In this study, we present a case of SBO, which was diagnosed and managed through laparoscopy successfully when a computed tomography (CT) scan was unable to reach a diagnosis. In doing this, laparotomy was avoided.

## Case presentation

A 60-year-old type 2 diabetic female presented to the emergency department with a four-day history of generalized abdominal pain and absolute constipation. The pain was continuous and aching in nature and was relieved by taking prokinetics. She had a past history of on-and-off constipation, which resolved with laxatives. There was no history of vomiting, fever, preceding diarrhea, weight loss or dyspepsia. There was no significant past surgical history.

An examination revealed a pulse of 94/min and BP 120/75 mm Hg. The abdomen was distended with sluggish bowel sounds. On deep palpation, there was mild generalized tenderness, but no sign of peritoneal or gall bladder inflammation was seen. A rectal examination revealed an empty rectum. She was managed in the emergency room on suspicion of acute abdomen. A nasogastric (NG) tube was passed and she was kept nil per os (NPO/nothing by mouth) and given intravenous (IV) fluids. The working diagnosis was sub-acute intestinal obstruction, which might have been due to colon cancer, pancreatitis, tuberculosis, or adhesions.

She was further investigated and the laboratory workup showed hemoglobin (Hb): 12.0 g/dl, total leukocyte count (TLC): 6800, amylase: 34 U/L, lipase: 19 U/L, and normal electrolytes. Her HbA1c was found to be 13.5%. Abdominal X-rays showed distended small bowel loops with no air fluid levels. She was admitted for conservative treatment but her distension increased the next day. CT of the abdomen and pelvis with oral and IV contrast revealed transition level at mid-ileum or distal jejunum with dilated proximal bowel. There was no obvious mass or lymph node enlargement. There was mild mesenteric congestion and minimal ascites. The CT scan gave no clue towards the cause of intestinal obstruction.

**Figure 1:**
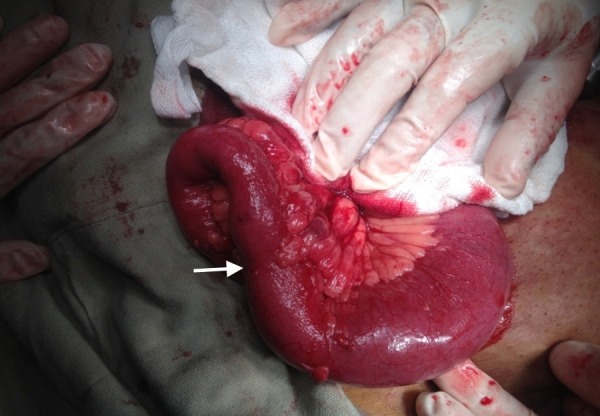
Per-Operative Findings: Distended Bowel Proximal to Stricture

Given her history, physical exam, and laboratory investigations, a minimally invasive diagnostic laparoscopy were planned. The patient was taken to the operation theater. Through the infra-umbilical incision, a Veress needle was inserted and pneumoperitoneum was created with great caution. Laparoscopy confirmed the CT scan findings. There were dilated small bowel loops up to the mid-ileum level and a stricture causing partial obstruction with no obvious mass and collapsed distal bowel. This is shown in Figure 1.

**Figure 2:**
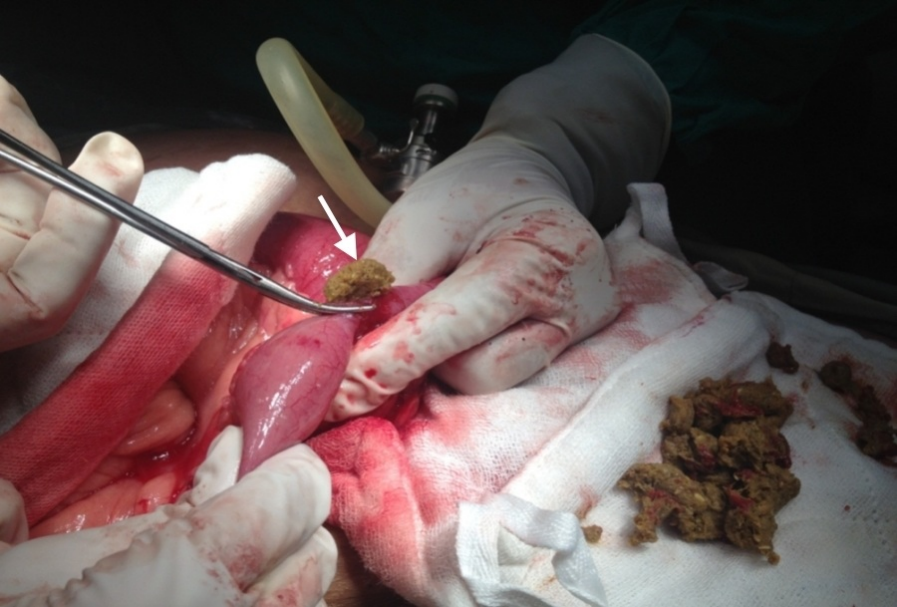
Removed Phytobezoars

A pathological bowel segment was brought outside through a small incision in the right iliac fossa. The stricture was causing partial obstruction. The bowel was opened for stricturoplasty and it revealed undigested phytobezoars causing obstruction. This is shown in Figure 2.

**Figure 3:**
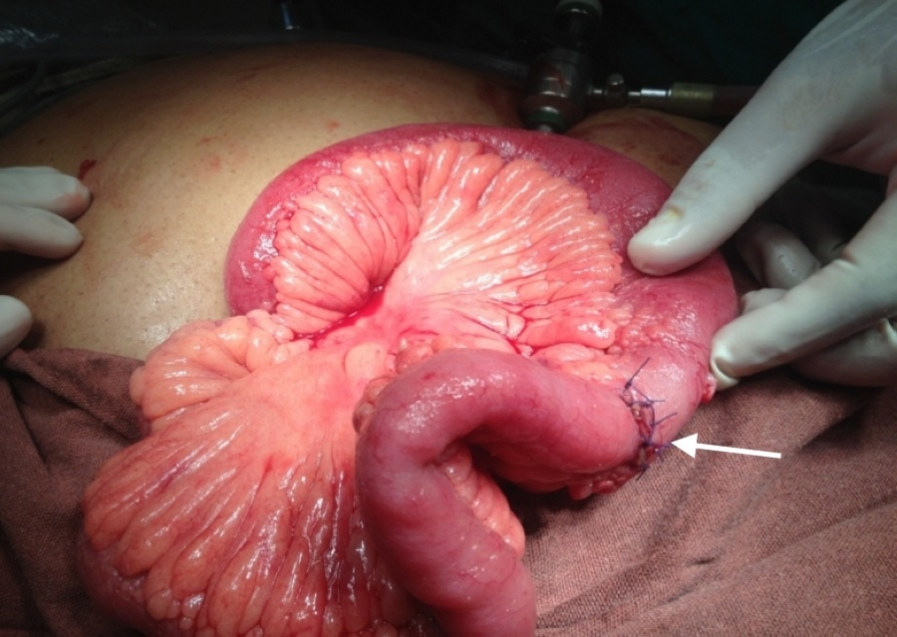
Extracorporeal Stricturoplasty

All undigested food particles were removed and extracorporeal stricturoplasty was done. This is shown in Figure 3. The wound was closed.

The postoperation recovery was uneventful, and on close questioning, the patient gave a history of intake of “spinach“ before this episode. She was discharged home on the third postoperative day in stable condition and was advised to increase water intake, modify diet (avoid stringy vegetables, high fiber foods, avoid persimmons) and chew food properly.

## Discussion

This case report signifies the role of laparoscopy in SBO due to phytobezoars, which gives an actual diagnosis with less morbidity as compared to laparotomy. A bezoar is a rare cause of SBO and can be difficult to diagnose and manage [4]. There is a range of different treatment options available to manage phytobezoars. Cellulase and papain enzymes have been used to reduce the size of phytobezoars [5]. Carbonated beverages such as Coca-Cola have been shown to be 91.3% effective in dissolving phytobezoars. Other medical treatment options include the administration of metoclopramide and N-acetylcysteine. Endoscopy has been used and has been proven to be quite successful when used for proximal bezoars. To make extraction easier, the bezoar can be broken down into smaller pieces via mechanical lithotripter, polypectomy snare, and drilling. Surgery, on the other hand, has been shown to be more effective for treating intestinal (distal) bezoars. Overall, surgery has a higher success rate (98.3%) as compared to endoscopy (89.7%) [6]. Surgery is also superior if there is evidence of intestinal obstruction or perforation.

Traditionally CT scans have been used to diagnose gastrointestinal bezoars. CT scans may demonstrate the bezoar as an intraluminal mass in the obstructed segment of the bowel. The mass may also appear mottled due to the presence of trapped air inside it [5]. As seen with any obstruction, the proximal gut can be dilated and the distal part collapsed. A CT scan can also detect multiple bezoars, and according to a study, the ability to delimit a bezoar and the diagnostic accuracy of a CT scan can be as high as 73-95% and 65-100%, respectively [7-8]. However, in some scenarios, such a highly accurate tool can fail to reach a diagnosis. Hence, to make an exact diagnosis, the bowel should be examined with the naked eye or through the eyes of a camera.

In recent times, laparoscopy has been used vastly in the diagnosis and management of small bowel obstruction due to experienced operators and increased availability. It can reveal the etiology of SBO well before the development of any serious complications, which in turn confers a favorable prognosis to the patient. As in our case, the patient underwent endoscopy immediately after the decompression of the gut and improved hydration status. The patient had an uneventful postoperative period with effective treatment of the SBO. Laparoscopy is a very safe and effective procedure with a strikingly lower rate of complications as compared to conventional laparotomy. Pathologies treated with laparoscopy instead of classic laparotomy incisions have shown to have a better postoperation outcome [9]. The comparison of the different surgical treatment options is shown in Table 1 [4, 10].

**Table 1:** Comparison of Outcomes of Laparotomy and Laparoscopy in the Management of Small Bowel Obstruction

Outcome	Laparoscopy	Laparotomy
Post-op complications	0 - 5.7%	0 - 18.6%
Post-op ileus	1.5 - 2.2 days	3 - 4.7 days
Hospital stay	2.5 - 6.6 days	5.8-16.8 days

## Conclusions

Phytobezoars should be considered in the differential diagnosis of SBO, especially in patients who have significant risk factors for the development of phytobezoars. Laparoscopy is a safe and effective diagnostic and therapeutic procedure in patients presenting with SBO secondary to phytobezoar. There is a need to formulate proper guidelines regarding the diagnosis and management of SBO due to phytobezoars.
